# The significant effects of cerebral microbleeds on cognitive dysfunction: An updated meta-analysis

**DOI:** 10.1371/journal.pone.0185145

**Published:** 2017-09-21

**Authors:** Xuanting Li, Junliang Yuan, Lei Yang, Wei Qin, Shuna Yang, Yue Li, Huimin Fan, Wenli Hu

**Affiliations:** Department of Neurology, Beijing Chaoyang Hospital, Capital Medical University, Beijing, China; Nathan S Kline Institute, UNITED STATES

## Abstract

**Objective:**

Accumulated data suggests that cerebral microbleeds (CMBs) play an important role in the decline of cognitive function, but the results remain inconsistent. In the current study, we aimed to investigate the association between CMBs and cognitive function, as well as the various effects of CMBs on different domains of cognition.

**Methods:**

We searched through the databases of PubMed, Embase, Cochrane Library, and ScienceDirect. After a consistency test, the publication bias was evaluated and a sensitivity analysis was performed with combined odds ratios (OR) and standardized mean difference (SMD) of CMBs.

**Results:**

A meta-analysis of 25 studies with 9343 participants total was conducted. Patients with CMBs had higher incidence of cognitive impairment (OR:3.5410; 95% confidence interval [CI] [2.2979, 5.4567], *p*<0.05) and lower scores of cognitive functions (SMD: -0.2700 [-0.4267, -0.1133], *p*<0.05 in Mini-Mental State Examination [MMSE] group and -0.4869 [-0.8902, -0.0818], *p*<0.05 in Montreal Cognitive Assessment [MoCA] group). Our results also indicated that patients with CMBs had obvious decline in cognitive functions, for instance, orientation (SMD: -0.9565 [-1.7260, -0.1869], *p*<0.05), attention and calculation (SMD: -1.1518 [-1.9553, -0.3484], *p*<0.05) and delayed recall (SMD: -0.5527 [-1.1043, -0.0011], *p* = 0.05).

**Conclusions:**

Our data suggested that CMBs might be an important risk factor for cognitive dysfunction, especially in the domains of orientation, attention and calculation and delayed recall functions. Prospective cohort studies with further investigations will be needed in larger samples.

## Introduction

According to the World Alzheimer Report (2015), the increasing prevalence of dementia will be one of the biggest global public health and social care challenges today and in the future. [1] Cerebral small vessel disease (CSVD) is an important clinical and pathological condition causing 20% of strokes worldwide, and one of the most common causes of vascular cognitive impairment (VCI) and mixed dementia. [2] Cerebral microbleeds (CMBs), detected by T2*-weighted gradient-recalled echo (GRE) or susceptibility-weighted imaging (SWI), have been recognized as an important manifestation and diagnostic marker of CSVD. Although CMBs have traditionally been considered as a part of clinical silence, a growing body of evidence has indicated that CMBs play a crucial role in the pathophysiology of VCI.

A population-based Rotterdam Scan Study shows that the incidence of CMBs was approximately 10% and CMBs rarely disappeared. [3] Studies from Asia also suggests that the presence of multiple CMBs, particularly multiple lobar CMBs, was associated with higher global neuropsychiatric burden on the Mini-Mental State Examination (MMSE) and Montreal Cognitive Assessment (MoCA). Such associations were more significant with CMBs located in deep areas and the increasing number of CMBs. [4–6] The Rotterdam Scan Study also suggested that presence of numerous microbleeds, especially in a strictly lobar location, was associated with worse performance on neuropsychological tests of information processing speed and motor speed. [7] According to a recent longitudinal study, participants with ≥3 CMBs had a substantial decline of global cognitive function, memory, and processing speed. [8] Over CMBs burden has a prognostic significance of cognitive impairment, however, there are also some controversies need to be clarified. For instance, one study from Netherland showed that CMBs were not associated with cognitive performances. [9] Another Dutch study also suggested that CMBs may have less featured influence on specific types of dementia such as frontotemporal lobar degeneration, progressive supranuclear palsy, and corticobasal degeneration. [10]

To date, there are two studies that performed meta-analysis to exam the relationship between CMBs and cognition. In 2013, a systematic review including 7 studies demonstrated that the presence of CMBs was significantly associated with cognitive impairment. [11] One year later, another meta-analysis also came to the similar conclusion that a higher number of CMB lesions and CMBs located in lobar regions, deep areas, basal ganglia, and thalamus regions were correlated with cognitive impairment, [12] but some limitations underlying in the above mentioned two meta-analyses need to be considered. For example, sample sizes of some included studies may be relatively small, and more high-quality studies with follow-ups needed to be further clarified. Such limitations may be due to the weakness of the included studies, the population selection bias and the only general cognition tests (MMSE and MoCA) with different sensitivity and specificity.

We performed an updated meta-analysis to renew the pooled estimates on the effects of CMBs on the general cognitive function and different domains of cognition. We also used the more appropriate selection criteria to minimize statistical and sample heterogeneity and more diversified scales such as composite Z scores based on neuropsychological tests to improve the accuracy of cognitive scores. [13, 14] As for the subgroup analysis, we also explored the association between CMBs and cognitive impairment according to different age groups.

## Material and methods

### Search methods

Studies were identified through the databases of PubMed, Embase, Cochrane Library, and ScienceDirect. The following were combined to yield our search outcome: (Dementia OR Alzheimer* OR Cognition[MeSH] OR Cognition OR Cognitive) AND (Microbleed* OR “Small vessel disease*” OR “Small vascular disease*” AND Cerebral OR Brain) AND Humans[MeSH]. This search was restricted only to articles published in English language. A reference list of reviewed articles related to the study was examined to substantiate the search, and for additional studies as well. The data sources were searched from Jan. 2000 to Dec. 2016.

### Inclusion and exclusion criteria

Studies were included if they fulfilled the following criteria: 1) case-control studies of CMBs and cognitive function of any age and sex, with participants divided into CMBs and non-CMBs groups; 2) CMBs defined as small, rounded, or ovoid, homogeneous, hypointense lesions with diameters less than 10 mm on T2*-GRE or SWI; disregarding the symmetrical hypointensities in the globi pallidi (calcification or iron deposition) and flow voids from cortical vessels; [15] 3) evaluating either global cognitive function in the aggregate such as MMSE and MoCA scales, or at least domains of cognitive function separately, including orientation, fluency, attention, memory, processing speed, executive function, naming, calculation, language, recall and so on.

Exclusion criterion: 1) a case report, review, meta-analysis, letter, editorial, treatment study; 2) participants with acute stroke and brain injury; 3) containing the crowd of multiple sclerosis, infection, epilepsy, cancer, serious mental illness, neural degeneration disease, or having taken drugs which effected on cognitive function within 24 hours; 4) repeated published studies with the same first author and data acquisition methods and similar patient characteristics, data analysis, and results; 5) studies with incomplete data.

The selection criteria were formulated to minimize statistical and sample heterogeneity.

### Data extraction and quality assessment

Two reviewers (XL and JY) independently examined all retrieved full texts that met the inclusion criteria. We extracted data including the first author, the publication data, MRI protocols, the number of CMBs and non-CMBs groups, mean age, and the number of male participants. We also extracted data on the presence and basic characteristics of CMBs, as well as on mean cognitive function scores and standard deviation (SD) obtained from MMSE, MoCA, and neuropsychological tests of CMBs and non-CMBs groups. If there were different adjusted models in the study, we chose the data with the most correction factors. When disagreement occurred in study classification, further discussion was undertaken to reach concordance. Disagreements were resolved through consultations with a third reviewer (WH).

Study quality was assessed using a modified version of the Newcastle-Ottawa Scale. A score of up to 9 points was assigned to each study based on the quality of group selection, comparability of groups and assessment of cognitive function.

## Statistical analyses

### Effect size and calculations

Collected data were analyzed using statistical software provided by the Cochrane Collaboration (RevMan 5.3). The odds ratio (OR) was calculated with corresponding 95% confidence interval (CI) for dichotomous variables. A 95% CI, excluding 1 or *p*<0.05, was considered statistically significant. Standardized mean difference (SMD) was calculated for continuous variables, in which a 95% CI, excluding 0 or *p*<0.05, was considered statistically significant.

### Assessment of bias

The funnel plot was used for the examination of publication bias (RevMan 5.3). To be more reliable, Begg’s tests and Egger’s tests were also conducted to quantify publication bias (Stata 12.0).

### Heterogeneity

Heterogeneity between study results was assessed using a standard I^2^ test. In general, according to the heterogeneity test selection combination method, a random-effect model (REM) was used when I^2^>50%. A fixed-effect model (FEM) was used when I^2^<50%.

### Sensitivity analysis

We used the fixed-effect model or excluded studies with low literature quality (NOS<8) to re-analyze the data and test the stability of this study.

## Results

### Description of studies

**Fig 1:**
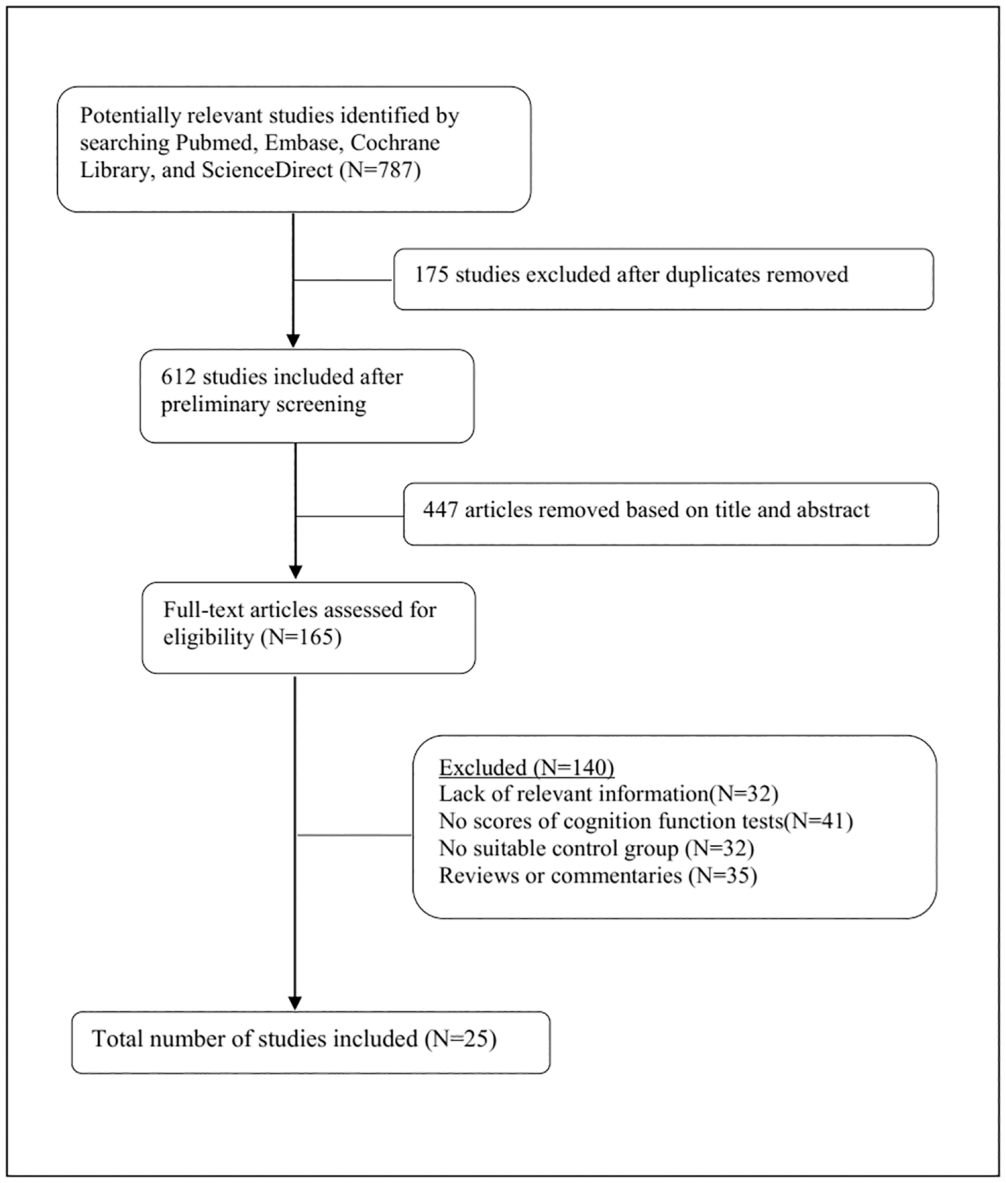
Flow diagram showing the progress of data collection.

We identified 787 potential studies from our initial electronic databases and reference lists, of which 175 studies were excluded after de-duplication. 447 studies were excluded after reviewing the titles and abstracts. The full texts of the remaining 165 studies were examined. Of those studies, 32 articles lacked relevant information. And 108 articles were excluded because the scores of cognition function tests were not reported, the studies lacked suitable control group, or the articles were reviews or commentaries. Eventually, 25 studies, comprising 1639 participants in CMBs group and 7704 in non-CMBs group, were all case-control studies and met all of the inclusion criteria and were included in the meta-analysis (Fig 1). Nineteen studies received a quality score of 8 out of 9. Five studies received a quality score of 7, and the remaining study received a quality score of 6 (Table 1).

**Table 1:** Characteristics of the studies included in the meta-analysis.

Reference	Country	Study Design	MRI Magnet	Cognitive Measurement	NOS	CMBs			Non-CMBs		
						Sample size, n	Male, n	Mean age, years	Sample size, n	Male, n	Mean age, years
Benedictus 2015[16]	Netherland	Case-control	1.0-T/1.5-T/3.0-T	MMSE	8	111	71	71.6±1.8	222	122	71.1±7.8
Chai 2016[17]	China	Case-control	3.0-T	MMSE	7	33	22	52.70±12.58	28	15	40.32±12.63
Doi 2015[18]	Japan	Case-control	1.5-T	MMSE	8	36	14	81.3±5.4	86	33	77.7±8.4
Fang 2013[19]	China	Case-control	3.0-T	MoCA/MMSE	8	41	24	70.6±5.2	91	47	71.4±5.9
Goos 2009[20]	Netherland	Case-control	1.0-T/1.5-T/3.0-T	MMSE	7	21	14	73±7	42	26	72±7
Goos 2010[21]	Netherland	Case-control	1.0-T	MMSE	8	31	16	69±9	223	117	66±10
Gregg 2015[22]	Room	Case-control	3.0-T	MMSE	8	21	14	87.6±2.8	34	19	86.2±2.5
Gregoire 2012 [23]	UK	Case-control	— —	NPT	7	9	6	65 (44–86)	17	10	62 (35–75)
Gustavsson 2015[24]	Sweden	Case-control	3.0-T	MMSE	8	25	15	73±5.5	182	69	72±4.6
Heringa 2014[25]	Netherland	Case-control	3.0-T	NPT/MMSE	8	26	12	80.7±6.9	41	16	76.4±7.3
Miwa 2014[26]	Japan	Case-control	— —	MMSE	8	113	78	68.1±8.8	401	224	67.3±8.1
Nakata-Kudo 2006[27]	Japan	Case-control	1.5-T	MMSE	7	8	3	74.4±7.1	42	14	74.6±7.7
Nardone 2011[28]	Austria	Case-control	1.5-T	MMSE	6	13	8	69.7(58–78)	20	13	69.2(55–79)
Qiu2010[29]	Sweden	Case-control	1.5-T	NPT	8	324	181	77.3±5.0	2794	1113	75.6 ±5.2
Ueda2016[30]	Japan	Case-control	3.0-T	MMSE	8	68	26	76.4±6.5	41	16	74±7.3
Valenti2016[31]	Italy	Case-control	1.5-T/3.0-T	MoCA/MMSE	8	41	24	75.3±6.4	111	63	75.6±6.8
van Es2011[32]	Netherland	Case-control	1.5-T	MMSE	8	106	— —	77±3	333	— —	— —
van Norden 2011[33]	Netherland	Case-control	1.5-T	MMSE	8	52	32	69.8±8	448	252	65.1±8.7
van2012[34]	Netherland	Case-control	1.0-T/1.5-T/3.0-T	MMSE	8	39	24	71±8	182	88	67±9
Werring2004[35]	UK	Case-control	1.5-T	NPT	7	25	18	67.6±11.9	30	20	67.2±10.4
Xu2017[36]	Singapore	Case-control	3.0-T	MoCA/MMSE	8	280	136	71.9±6.8	522	233	69.4±6.4
Yakushiji 2008[5]	Japan	Case-control	1.5-T	MMSE	8	35	— —	57.6 (52.3–63.6)	483	— —	56.8(50.1–63.8)
Yakushiji 2012[6]	Japan	Case-control	1.5-T	MMSE	8	98	— —	63 (58–67)	1181	— —	58 (50–65)
Yamashiro 2014[37]	Japan	Case-control	1.5-T	MMSE	8	48	— —	70.0 ± 8.0	100	— —	73.5 ±8.6
Zhang2013[38]	China	Case-control	3.0-T	MoCA	8	35	— —	Number of aged ≥65 = 9	50	— —	Number of age≥65 = 30

CMBs = cerebral microbleeds; MMSE = Mini-Mental State Examination; MoCA = Montreal cognitive assessment; NPT = neuropsychological tests; NOS = Newcastle-Ottawa Scale

### Heterogeneity test and synthesized efficacy

#### Incidence of cognitive dysfunction

**Fig 2:**
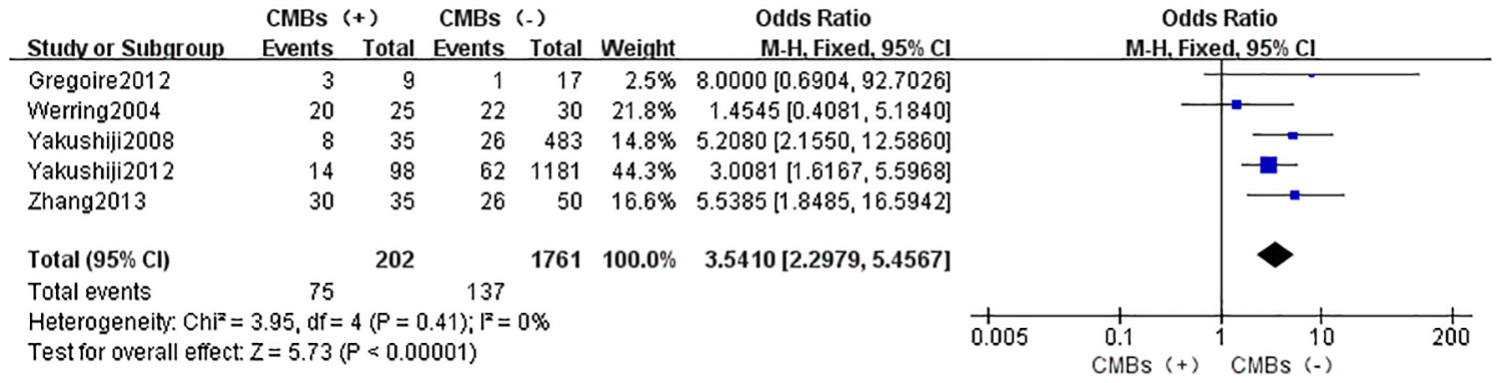
Meta-analysis of incidence of cognitive dysfunction in CMBs versus non-CMBs. CMBs = cerebral microbleeds; Fixed = the fixed-effect model; 95% CI = 95% confidence interval.

Five studies were eligible for comparing the incidence of cognitive dysfunction in CMBs versus non-CMBs patients. The total number of participants was 1963 (CMBs group:202, non-CMBs group:1761). A fixed-effect model was used for meta-analysis with heterogeneity I^2^ of 0%. This analysis showed a significantly higher rate of cognitive impairment in CMBs group with OR: 3.5410[2.2979, 5.4567] (*p*<0.05) when compared to non-CMBs group (Fig 2).

#### Comparison of cognitive function assessment

**Fig 3:**
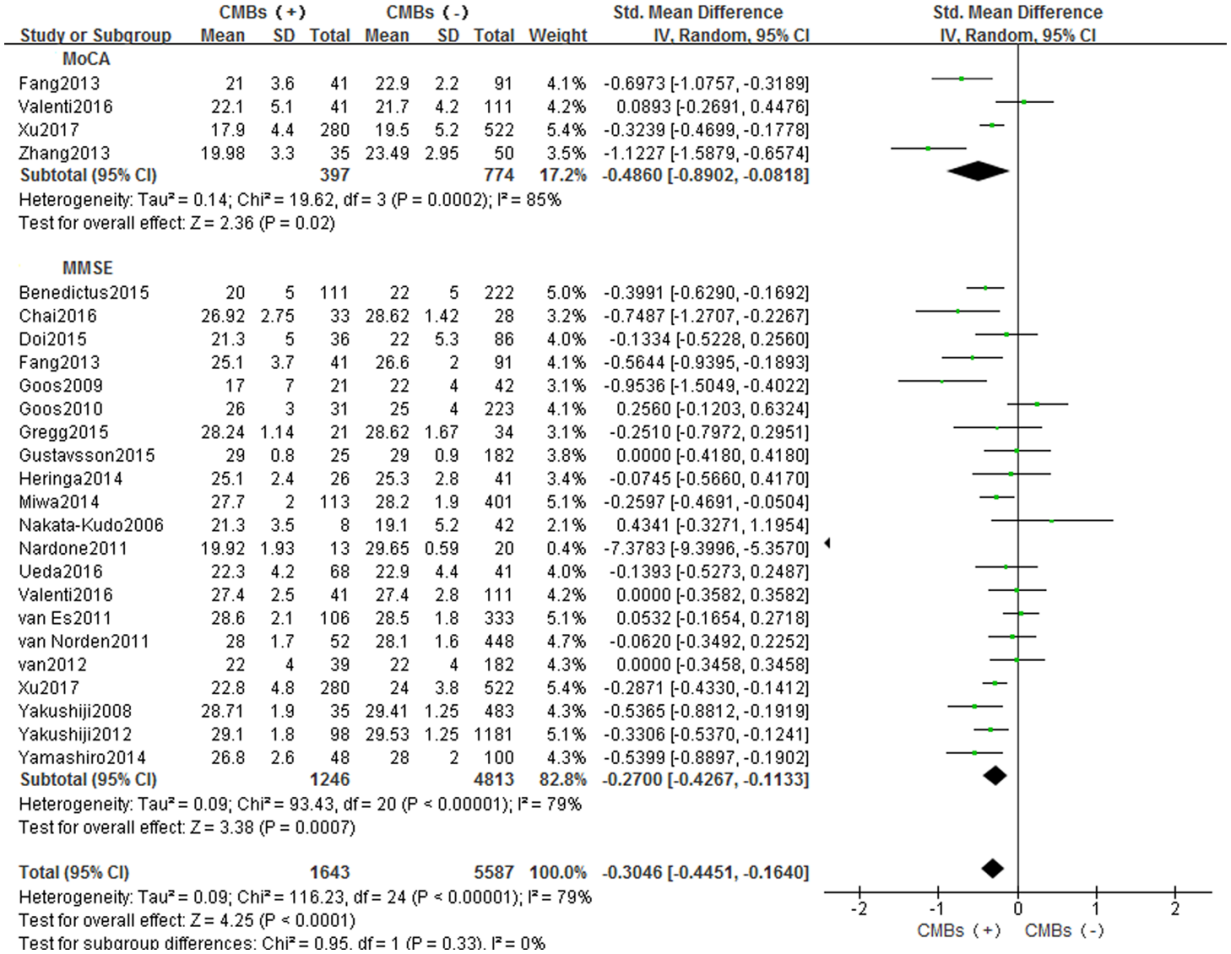
Meta-analysis of cognitive function assessment in CMBs versus non-CMBs based on MMSE and MoCA. CMBs = cerebral microbleeds; SD = standard deviation; Random = the random-effect model; 95% CI = 95% confidence interval; MMSE = Mini-Mental State Examination; MoCA = Montreal Cognitive Assessment.

A total of 6154 participants (CMBs group:1291 and non-CMBs group:4863) from 21 studies were eligible. A random-effect model was used with I^2^ of 79%. This finding suggested that CMBs patients had an impaired cognitive function compared with non-CMBs patients with SMD: –0.3046 [–0.4451, –0.1640] (*p*<0.05). In the subgroup analysis based on cognitive scales, both values suggested a lower cognitive function of CMBs patients with SMD: –0.2700 [–0.4267, –0.1133] (*p*<0.05) in MMSE group and –0.4860 [–0.8902, –0.0818] (*p*<0.05) in MoCA group. (Fig 3).

A subgroup analysis of MMSE with 16 studies [5,6,16,17,19–22,24,26–28,30,31,33,37] was carried out and divided into different age groups. A random-effect model was used with I^2^ of 82%, and SMD: -0.3498 [-0.5624, -0.1372] (*p*<0.05). And the SMD was -0.5111 [-0.9229, -0.0993] (*p*<0.05) in the mean age<70 group and -0.3027 [-0.5282, -0.0773] (*p*<0.05) in the mean age<80 group (Table 2, S1 Fig).

**Table 2:** Subgroup analysis of cognitive function assessment in CMBs versus non-CMBs based on age of MMSE.

Group	CMBs (+)	CMBs (-)	SMD, Random, 95%*CI*	*P*-value
Mean age<70	375	2784	-0.5111 [-0.9229, -0.0993]	<0.05
Mean age<80	363	831	-0.3027 [-0.5282, -0.0773]	<0.05
Mean age<90	21	34	-0.2510 [-0.7972, 0.2951]	0.37
Total	759	3649	-0.3498 [-0.5624, -0.1372]	<0.05

CMBs = cerebral microbleeds; SMD = standardized mean difference; Random = the random-effect model (I^2^ = 82%); MMSE = Mini-Mental State Examination

#### Comparison of different cognitive domains

**Fig 4:**
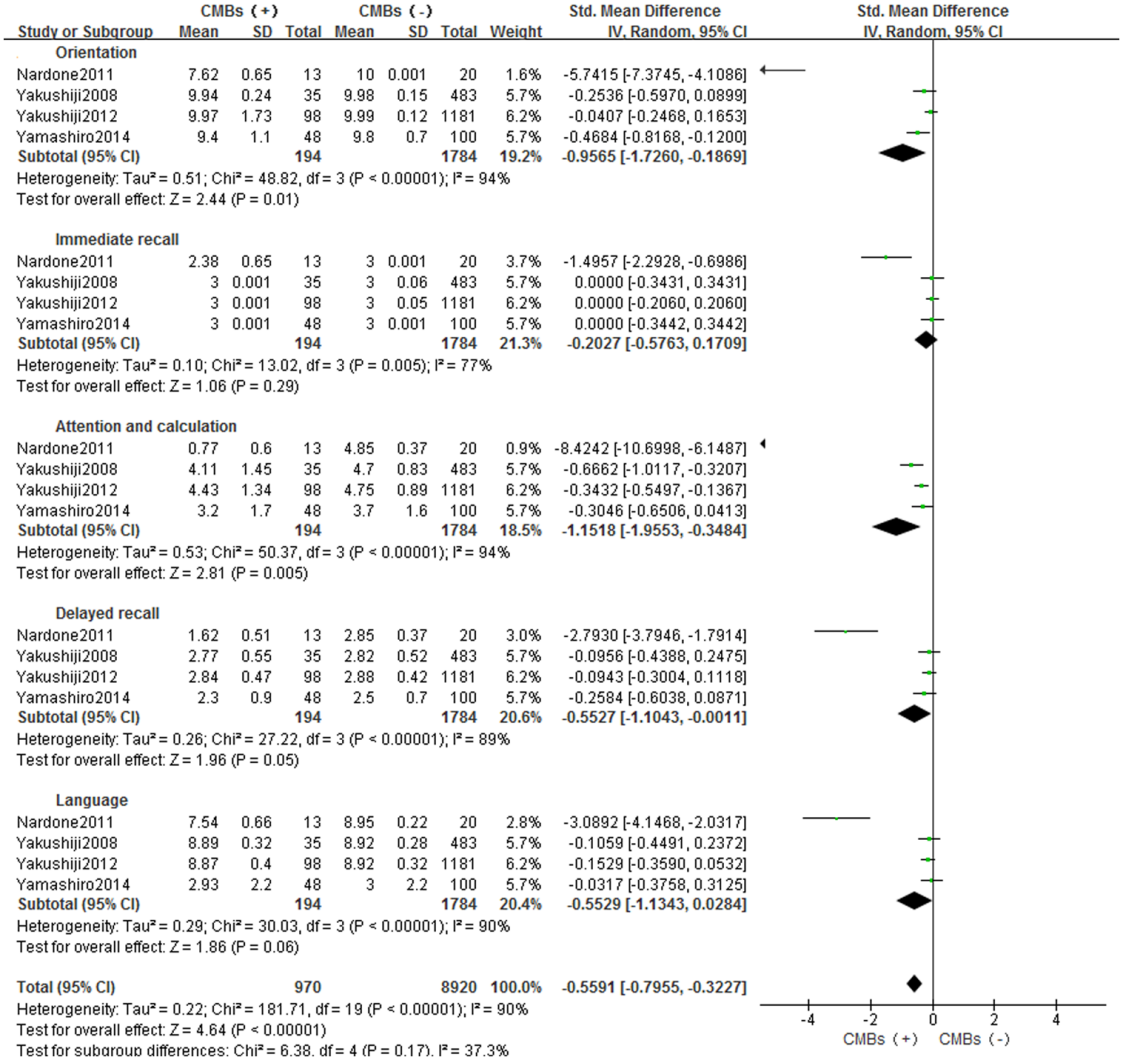
Meta-analysis of different cognitive domains in CMBs versus non-CMBs based on MMSE. CMBs = cerebral microbleeds; SD = standard deviation; Random = the random-effect model; 95% CI = 95% confidence interval; MMSE = Mini-Mental State Examination.

In four studies, data are represented as mean (SD) of each cognitive domain. A random-effect model was used with I^2^ of 90%, and SMD: -0.5591 [-0.7955, -0.3227] (*p*<0.05). Compared with the control group, patients with CMBs had impaired orientation (SMD: -0.9565[-1.7260, -0.1869], *p*<0.05), attention and calculation (SMD: -1.1518[-1.9553, -0.3484], *p*<0.05) and delayed recall (SMD: -0.5527[-1.1043, -0.0011], *p* = 0.05) functions (Fig 4).

**Fig 5:**
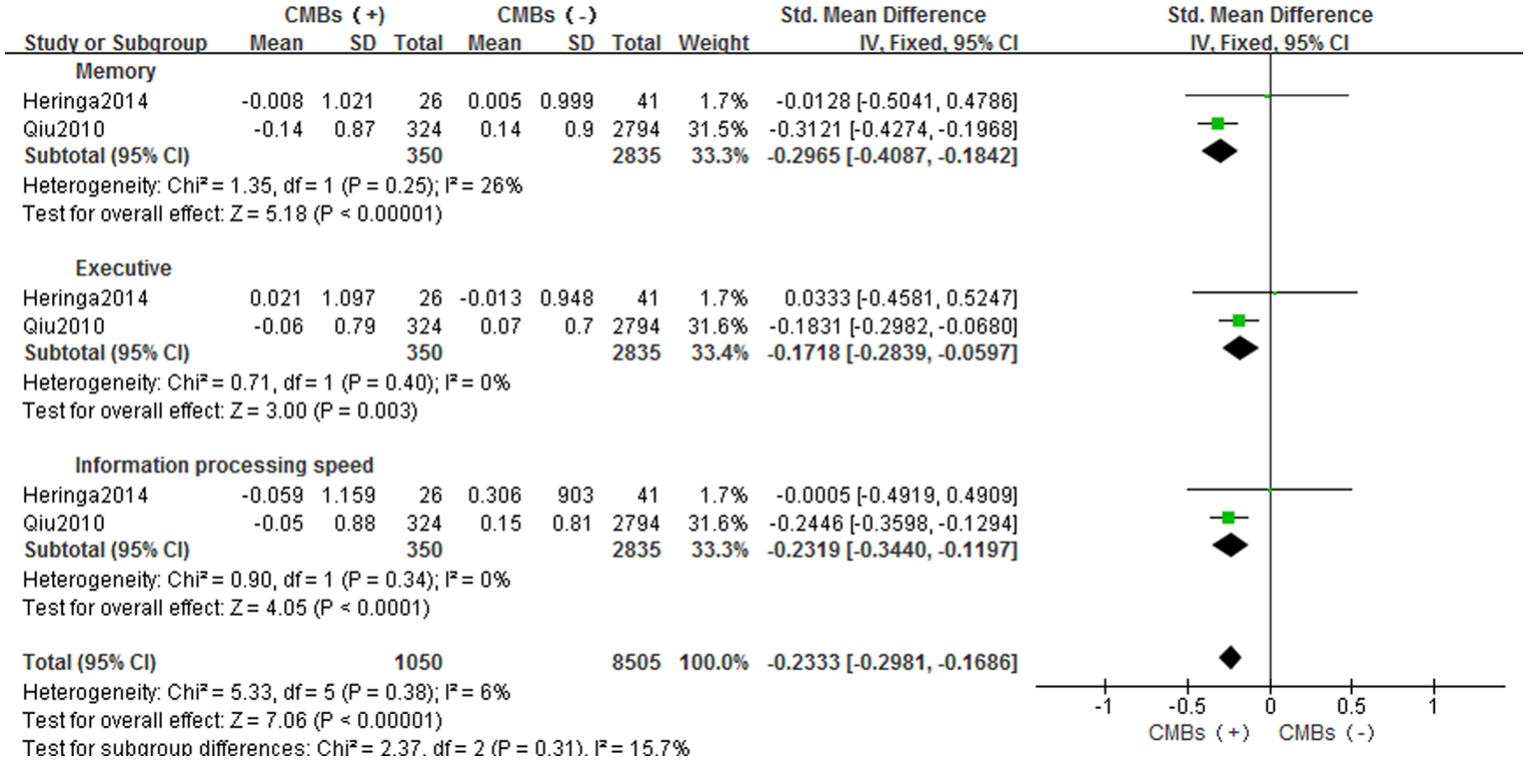
Meta-analysis of different cognitive domains in CMBs versus non-CMBs based on neuropsychological tests in Z scores. CMBs = cerebral microbleeds; SD = standard deviation; Fixed = the fixed-effect model; 95% CI = 95% confidence interval.

In two studies, the composite score for each cognitive domain was computed by converting raw scores to standardized Z scores and averaging them across the neuropsychological tests for each domain. A fixed-effect model was used with I^2^ of 6%, and SMD: -0.2333 [-0.2981, -0.1686] (*p*<0.05). Compared with the control group, patients with CMBs had impaired memory (SMD: -0.2965[-0.4087, -0.1842], *p*<0.05), executive (SMD: -0.1718[-0.2839, -0.0597], *p*<0.05) and information processing speed (SMD: -0.2319[-0.3440, -0.1197], *p*<0.05) functions (Fig 5).

### Publication bias and sensitivity analysis

**Fig 6:**
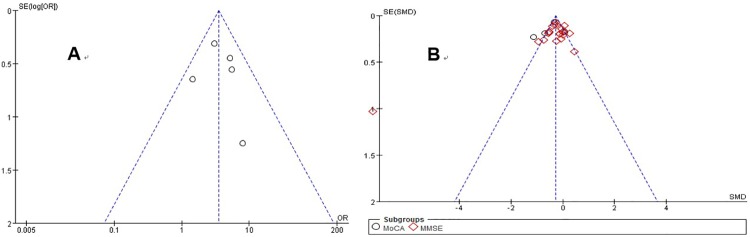
**Funnel plot of comparison: (A) Incidence of cognitive impairment in CMBs and non-CMBs patients. (B) Comparison of cognitive function assessment between CMBs versus non-CMBs**. CMBs = cerebral microbleeds; OR = odds ratio; SMD = standardized mean difference; MMSE = Mini-Mental State Examination; MoCA = Montreal Cognitive Assessment.

The funnel plot for studies on the incidence of cognitive impairment was symmetrical (Fig 6A). Both Egger’s (*p* = 0.806) and Begg’s (*p* = 0.694) tests showed that there was no significant publication bias for these studies. Similar results were obtained for studies on cognition scores (MMSE and MoCA). The funnel plots indicated an absence of publication bias (Fig 6B), as illustrated by both Egger’s (*p* = 0.252) and Begg’s (*p* = 0.180) tests.

The data were re-analyzed using the Random-effect model for studies on the incidence of cognitive impairment. And the stability of the results reflected the reliability of the combined results to a certain extent with OR: 3.5596[2.3234, 5.4535] (*p*<0.05).

Similar results were obtained for studies on cognition scores on MMSE and MoCA with SMD: -0.2721 [-0.3304, -0.2137] (*p*<0.05) using the fixed-effect model. After excluding the four studies with low literature quality (NOS<8), the results showed that CMBs patients had an impaired cognitive function compared with non-CMBs patients (SMD: -0.2484 [-0.3562, -0.1407], *p*<0.05), and no significant changes were obtained before and after the elimination.

## Discussion

In our present meta-analysis, we have confirmed that CMBs might be an important risk factor for cognitive dysfunction, especially in the domains of orientation, attention and calculation and delayed recall functions. Furthermore, the composite Z scores also showed that patients with CMBs performed impaired memory, executive and information processing speed functions compared to controls.

CMBs are considered to be important neuroimaging markers of CSVD. CMBs have been recognized to be disorders of brain damage caused by both vascular factors and amyloid pathologic mechanisms. [39] To date, whether their presence of CMBs is associated with cognitive decline in the general population remains poorly understood. Recently, a meta-analysis from China found that patients with CMBs had higher incidence of cognitive impairment but with some limitations, i.e. their data base include scores of MMSE. [12] MMSE scale has a less sensitivity for the diagnosis of mild cognitive impairment (MCI) than MoCA scale and adopting a cutoff ≥17 on MoCA could improve the sensitivity for early diagnosis. [40] Another point is that MMSE scale does not include executive function items. In order to resolve such problem, we collected studies using neuropsychological tests with Z scores, which were more diversified and more comprehensive to measure the function of different cognitive domains. Notably, a previous longitudinal study also used Z scores in the assessment of cognitive function, and their results supported our conclusion that numerous CMBs were the risk factor of the steeper decline of memory and speed. [8] In addition, as an important factor both for CMBs and cognition, age stratification has never been discussed about the effect of CMBs on cognition in the previous meta-analysis. Our current study made a compilation of previous studies, and different age based on the scores of MMSE were considered. Our analysis showed that patients with CMBs had an obvious cognitive decline in the mean age<70 group and<80 group, while there was no significant difference in the mean age<90 group. However, more studies based on larger population will be needed to explore our conclusion in the future.

As an updated meta-analysis, strengths of our study are the much larger sample size of case-control studies, the use of an extensive neuropsychological test battery, and compound scores of neuropsychological results of specific cognitive domains. Furthermore, in order to explore the effects of age on the association between CMBs and cognition, we also performed subgroup analysis according to the age.

As for the limitations, sample sizes, measure scales and measurement bias of the included studies mainly limit our meta-analysis. First, as for sample sizes, some studies [6, 16, 26, 29, 32, 36] involving a relatively large number of participants with CMBs were included, while others were dozens of participants. So, it was likely to cause selection bias due to different sample sizes. Second, variant population with different age, male rate, educational level and basic diseases may be the causes of heterogeneity in our meta-analysis. Third, different magnet strengths of MRI may lead to the heterogeneity in the numbers of detected CMB lesions. Fourth, only four studies [5, 6, 28, 37] included a detailed analysis about the association between CMBs and cognitive domains based on MMSE, which meant that the impact of CMBs on different cognitive domains remained controversial. Last, the non-randomised meta-analysis applied here with non-blinded identification of CMB lesions or assessment of cognitive function was easily affected by confounding and bias.

## Conclusion

In summary, patients with CMBs have higher incidence of cognitive impairment, particularly in orientation, attention and calculation, delayed recall functions, memory, executive function and information processing speed. These concepts of CMBs might provide theoretical references for establishing an early detection, prevention and treatment of VCI. Future work may focus on prospective cohort studies with larger population and the specific treatments to reduce the formation of VCI or to resolve such lesions of CMBs.

## Supporting information

S1 File PRISMA 2009 checklist. (DOC)

S2 File PRISMA 2009 flow diagram. (DOC)

S1 Fig Subgroup analysis of cognitive function assessment in CMBs versus non-CMBs based on age of MMSE. CMBs = cerebral microbleeds; SD = standard deviation; SMD = standardized mean difference; Random = the random-effect model; MMSE = Mini-Mental State Examination.
(TIF)
