# Supplementary material for: The significant effects of cerebral microbleeds on cognitive dysfunction: An updated meta-analysis
Source: PLoS One. 2017 Sep 21;12(9):e0185145. doi: 10.1371/journal.pone.0185145 (PMC5608335; doi:10.1371/journal.pone.0185145)
Supplement: S2 File — (DOC) [file pone.0185145.s002.doc]

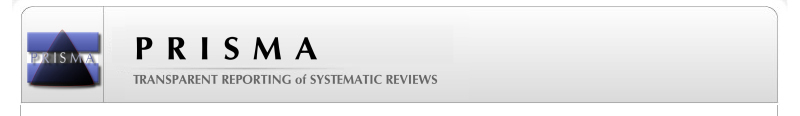
**PRISMA 2009 Flow Diagram**

**Screening**

**Included**

**Eligibility**

**Identification**

Potentially relevant studies identified by searching Pubmed, Embase, Cochrane Library, and ScienceDirect (N=787)

175 studies excluded after duplicates removed

447 articles removed based on title and abstract

612 studies included after preliminary screening

Full-text articles assessed for eligibility (N=165)

Excluded (N=140)

Lack of relevant information(N=32)

No scores of cognition function tests(N=41)

No suitable control group (N=32)

Reviews or commentaries (N=35)

Total number of studies included (N=25)
