# Supplementary figures and images for: The significant effects of cerebral microbleeds on cognitive dysfunction: An updated meta-analysis
Source: PLoS One. 2017 Sep 21;12(9):e0185145. doi: 10.1371/journal.pone.0185145 (PMC5608335; doi:10.1371/journal.pone.0185145)

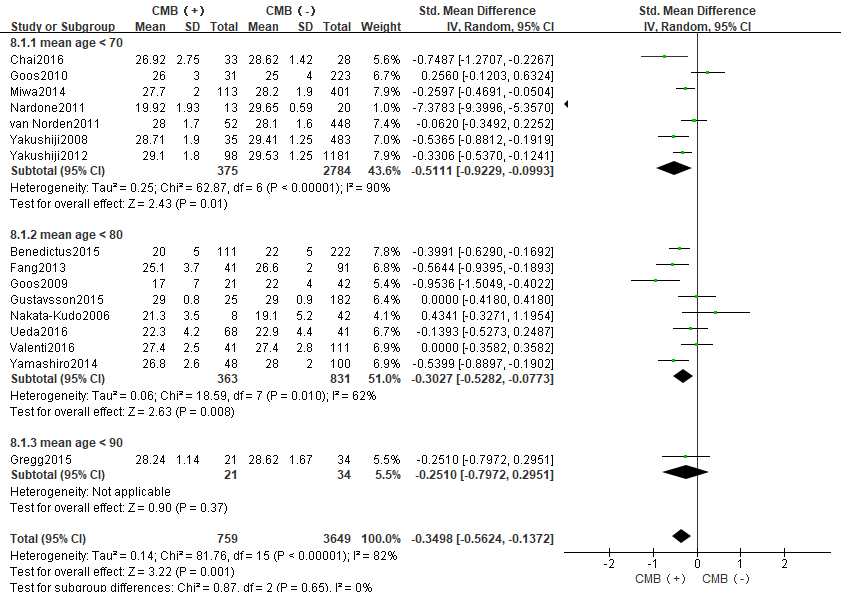

Supplement: S1 Fig — CMBs = cerebral microbleeds; SD = standard deviation; SMD = standardized mean difference; Random = the random-effect model; MMSE = Mini-Mental State Examination. (TIF) [file pone.0185145.s003.tif]
